# Supplementary material for: Bio-Potency and Molecular Docking Studies of Isolated Compounds from Grewia optiva J.R. Drumm. ex Burret
Source: Molecules. 2021 Apr 1;26(7):2019. doi: 10.3390/molecules26072019 (PMC8036409; doi:10.3390/molecules26072019)
Supplement: Supplementary file 1 [file molecules-26-02019-s001.pdf]

Supplementary Material

Bio-potency and molecular docking studies of isolated compounds from *Grewia optiva* J.R. Drumm. ex Burret

Wasim Ul Bari[1], **Najeeb Ur Rehman**[2], **Ajmal Khan**[2], **Ye Yuan**[3], **Mark A. T. Blaskovich**[3], **Zyta M. Ziora**[3], Muhammad Zahoor [1, 4], Sumaira Naz [4], **Riaz Ullah**[5], **Siddique A. Ansari**[6], **Hafiz Majid Mahmood**[7] **and Ahmed Bari**[8], **and Ahmed Al-Harrasi**[2]

1 Department of Chemistry, University of Malakand, Chakdara, Dir Lower, 18800 KPK, Pakistan. Email: wasimouch4080@gmail.com
2 Natural and Medical Sciences Research Center, University of Nizwa, Birkat Al Mauz, Sultanate of Oman. Email: najeeb@unizwa.edu.om, ajmalchemist@yahoo.com
3 Centre for Superbug Solutions, Institute for Molecular Bioscience, The University of Queensland, Brisbane, Queensland, 4072, Australia 4072. Email: ye.yuan1@uq.net.au, m.blaskovich@imb.uq.edu.au, z.ziora@uq.edu.au
4 Department of Biochemistry, University of Malakand, Chakdara, Dir Lower, 18800 KPK, Pakistan. Email: mohammadzahoorus@yahoo.com sumaira.biochem@gmail.com
5 Department of Pharmacognosy (MAPPRC), College of Pharmacy, King Saud University, Ri-yadh, Saudi Arabia. Email: rullah@ksu.edu.sa
6 Department of Pharmaceutical Chemistry, College of Pharmacy, King Saud University, Riyadh, Saudi Arabia. Email: sansari@ksu.edu.sa
7. Department of Pharmacology, College of Pharmacy, King Saud University, Riyadh, Saudi Arabia. Email: harshad@ksu.edu.sa
8 Department of Soil Sciences, College of Food and agriculture Sciences, King Saud University PO box 2460, Riyadh 11451, Saudi Arabia. Email: abari@ksu.edu.sa
* Correspondence: author: Dr. Muhammad Zahoor, Email: mohammadzahoorus@yahoo.com

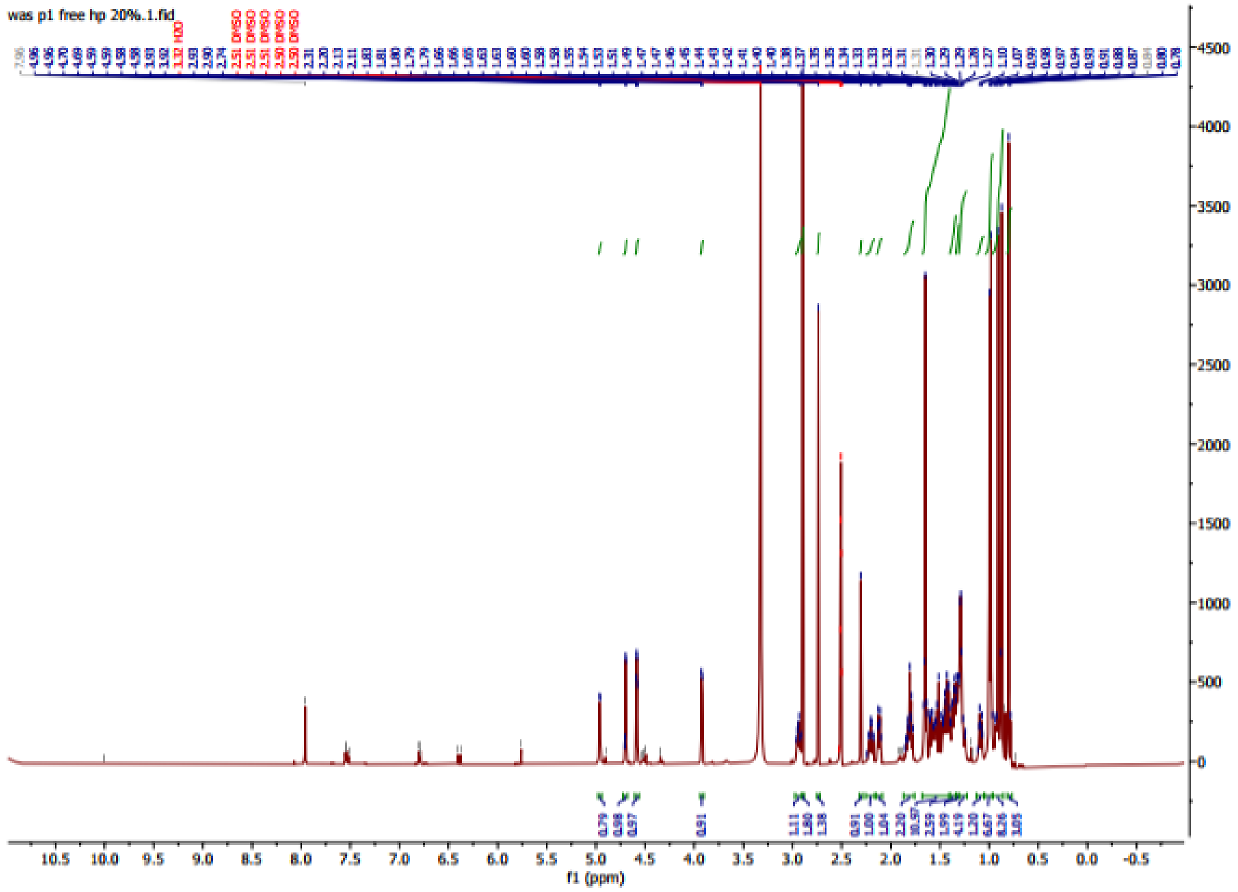

Figure S1: Proton NMR of compound **IX**

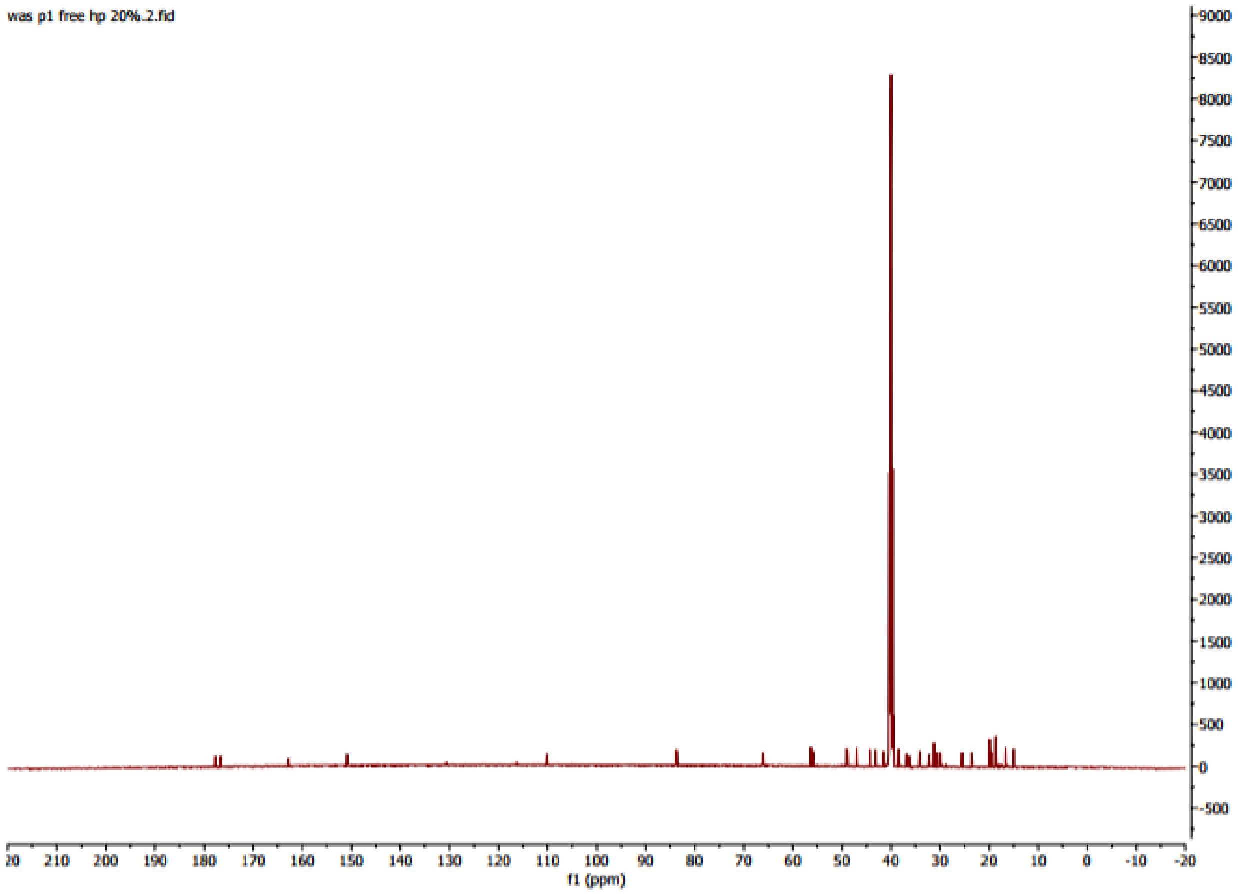

Figure S2: Carbon-13 NMR of compound **IX**

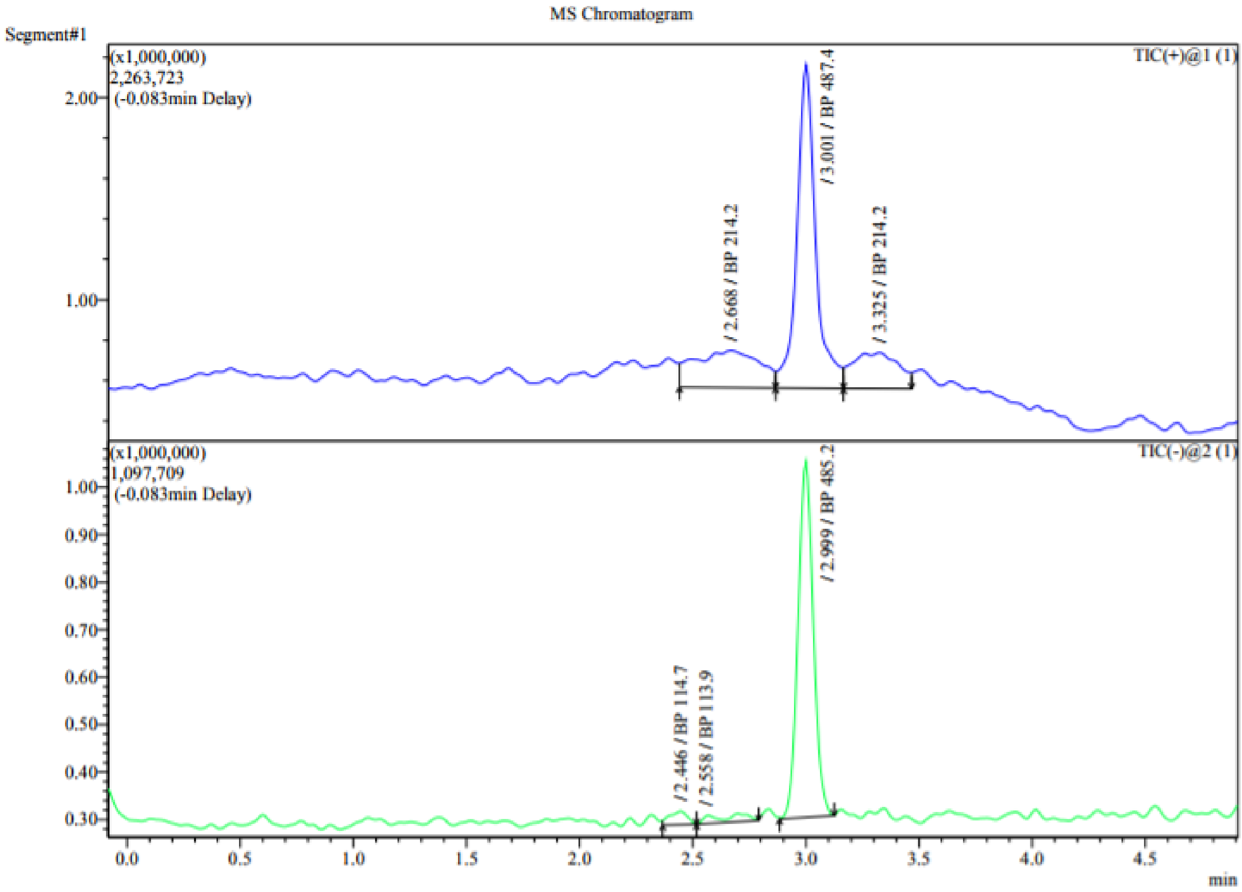

Figure S3: LC-MS chromatogram of compound **IX**

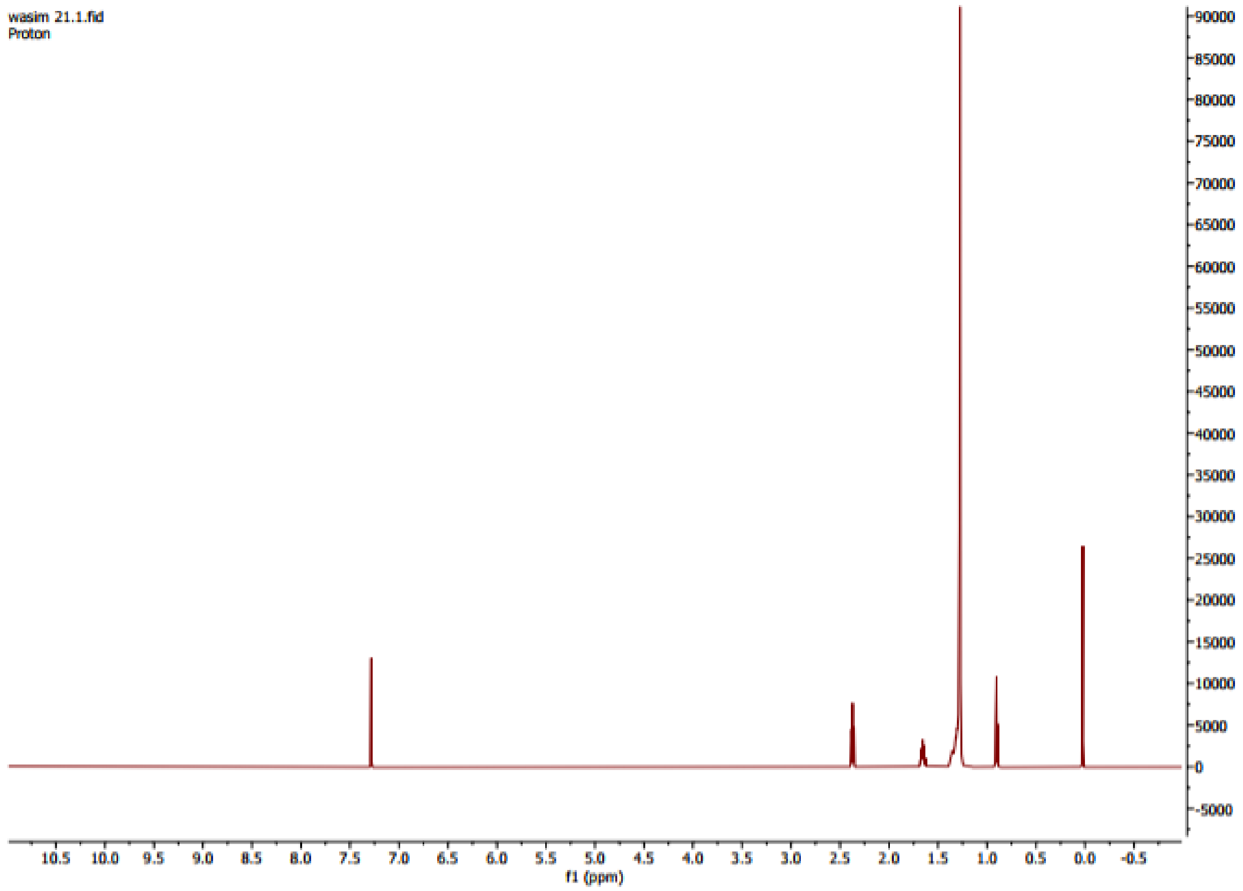

Figure S4: Proton NMR of compound **X**

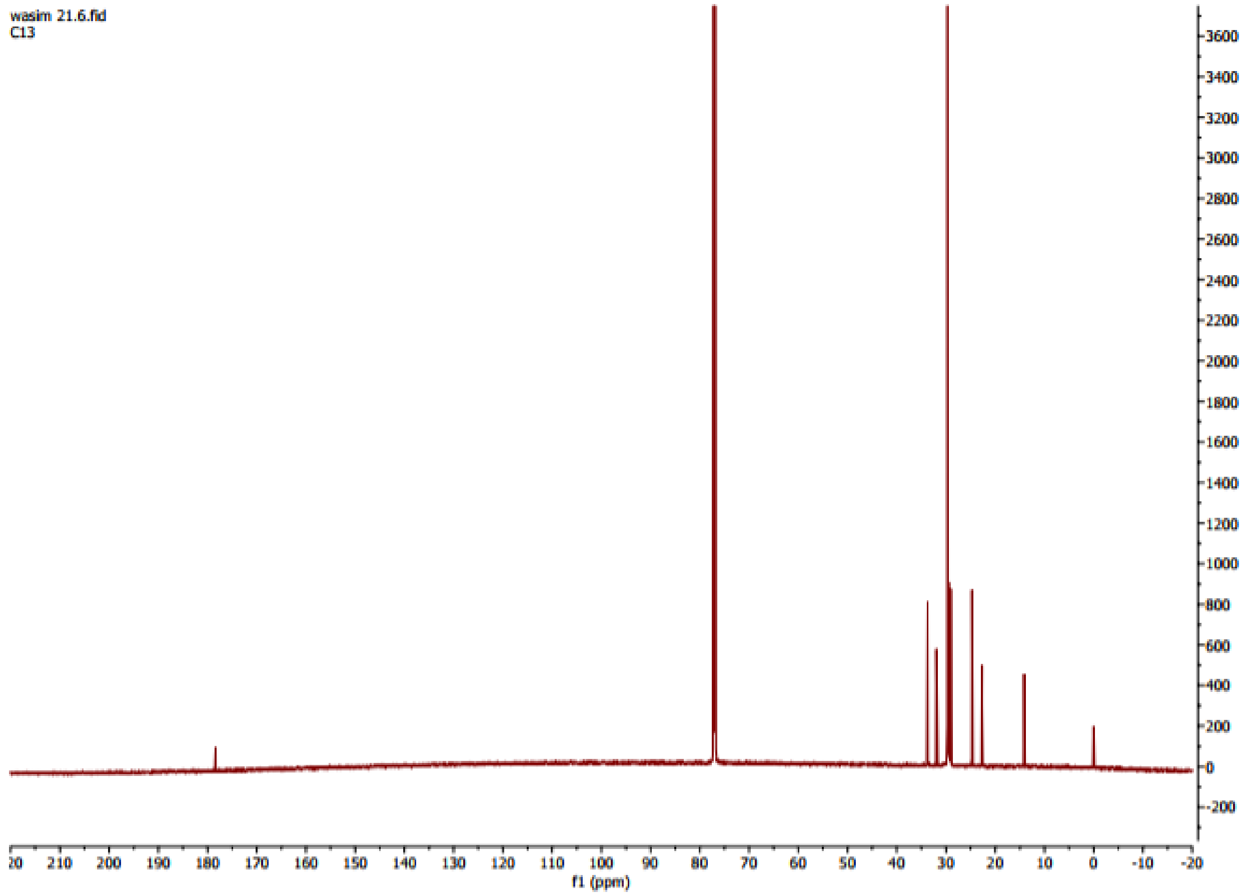

Figure S5: Carbon-13 NMR of compound **X**

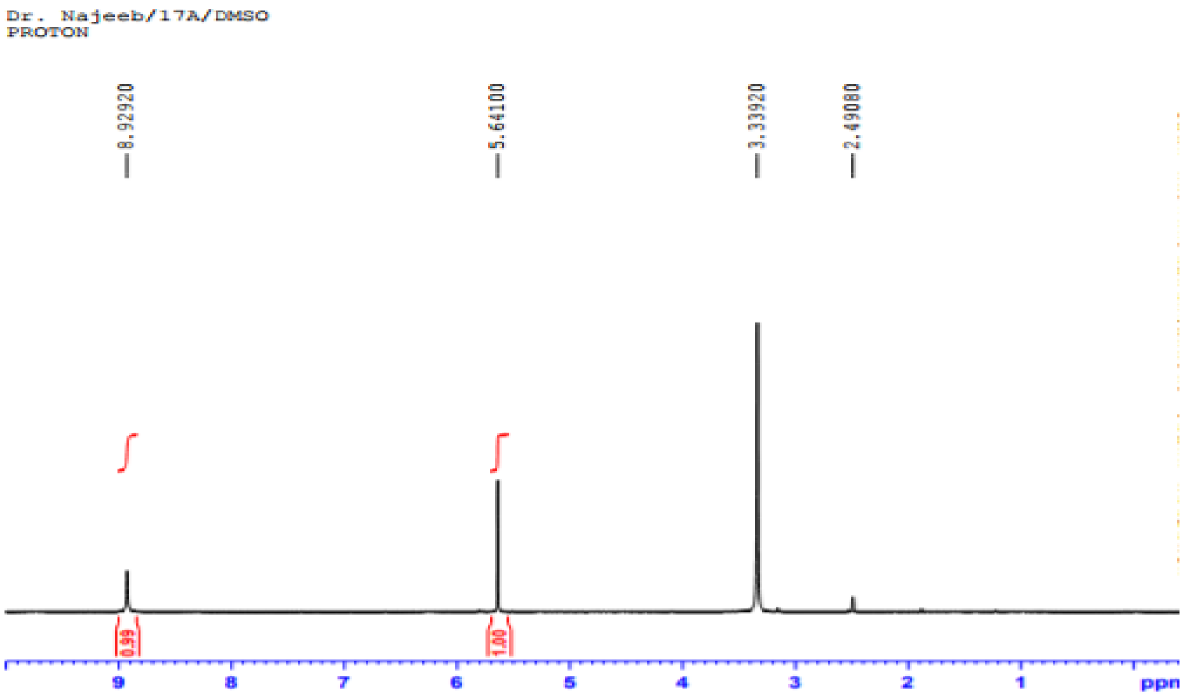

Figure S6: Proton NMR of compound **XI**

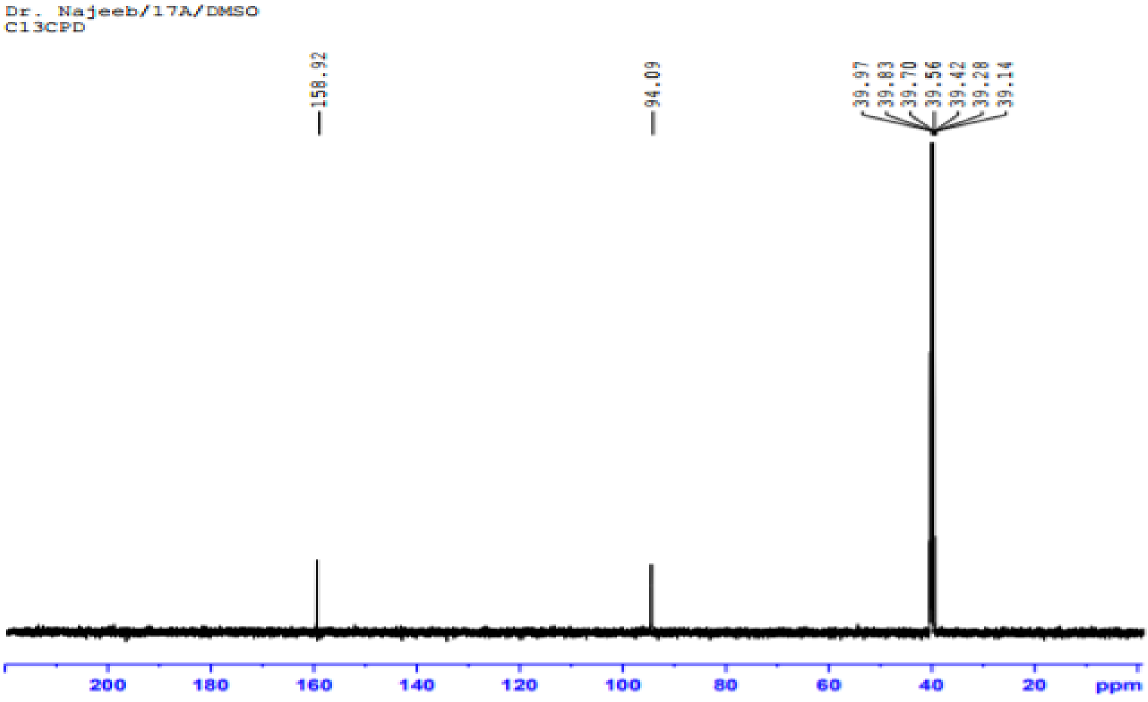

Figure S7: Carbon-13 NMR of compound **XI**

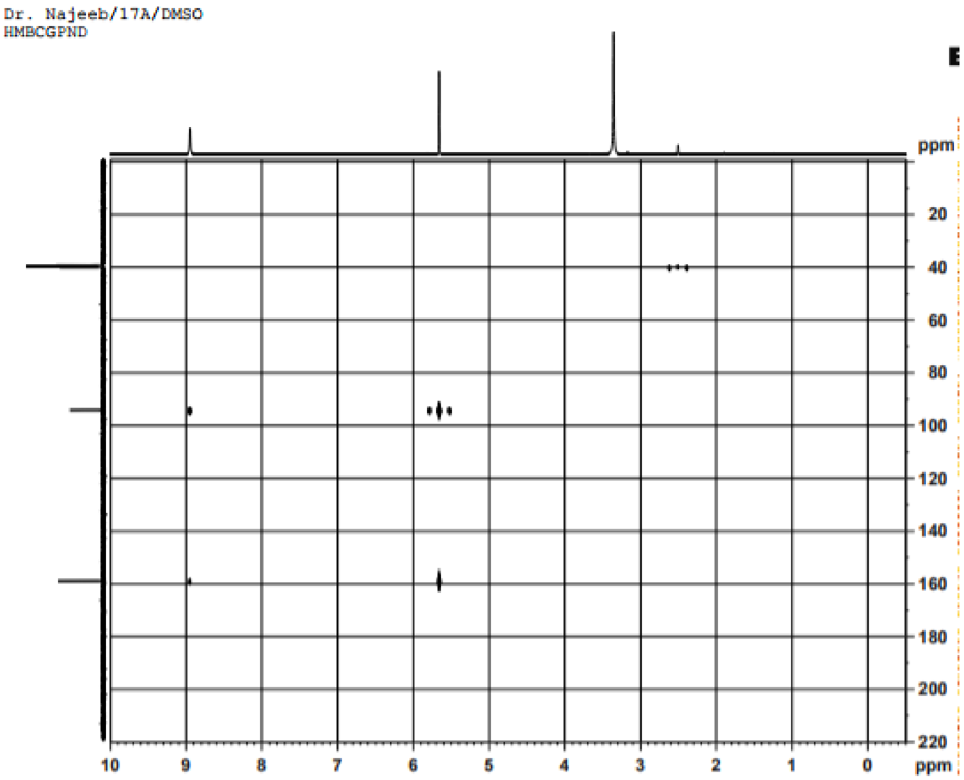

Figure S8: HMBC of compound **XI**

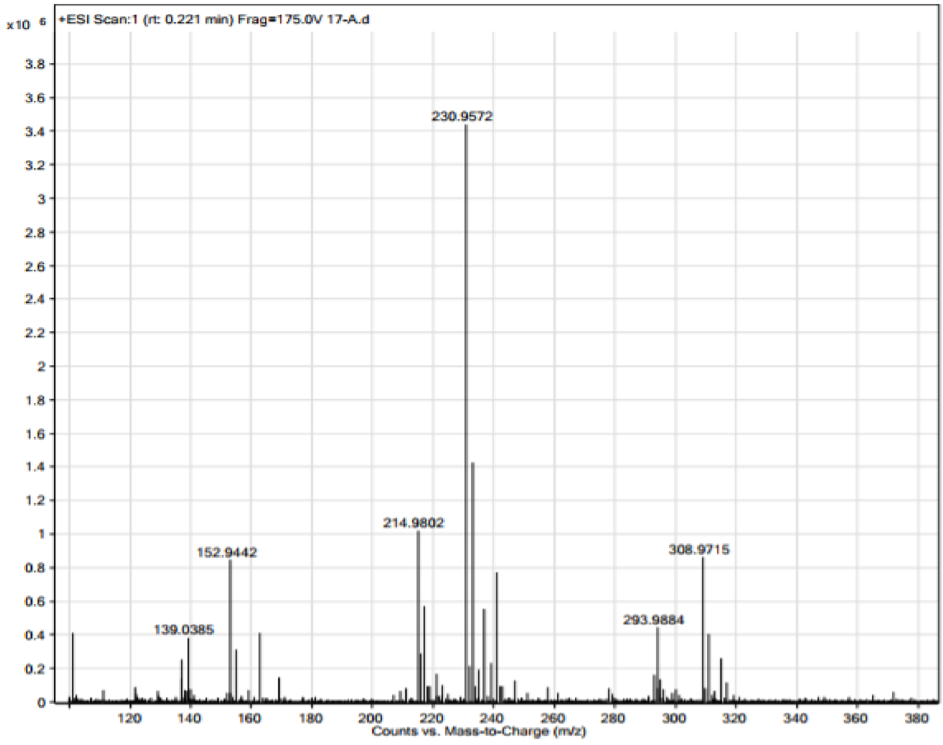

Figure S9: Mass spectra of compound **XI**

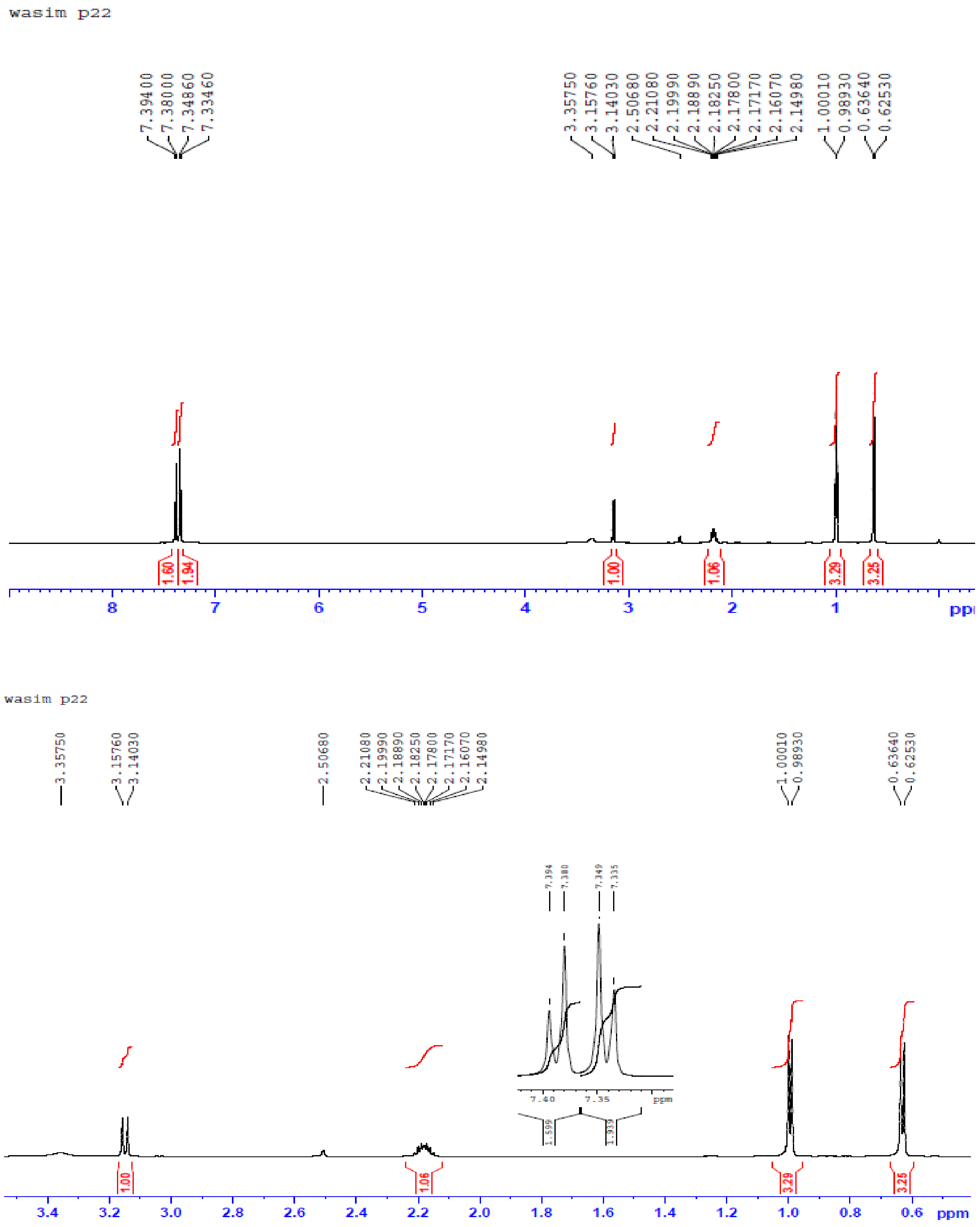

Figure S10: Proton NMR of compound **XII**

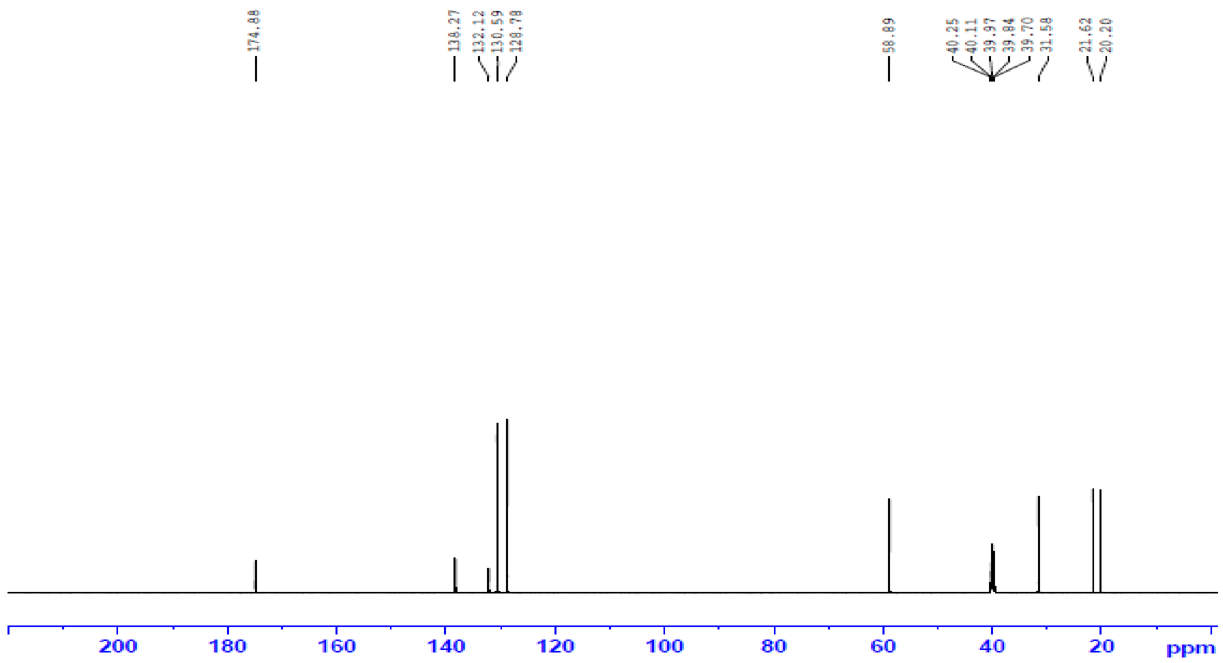

Figure S11: Carbon-13 NMR of compound **XII**

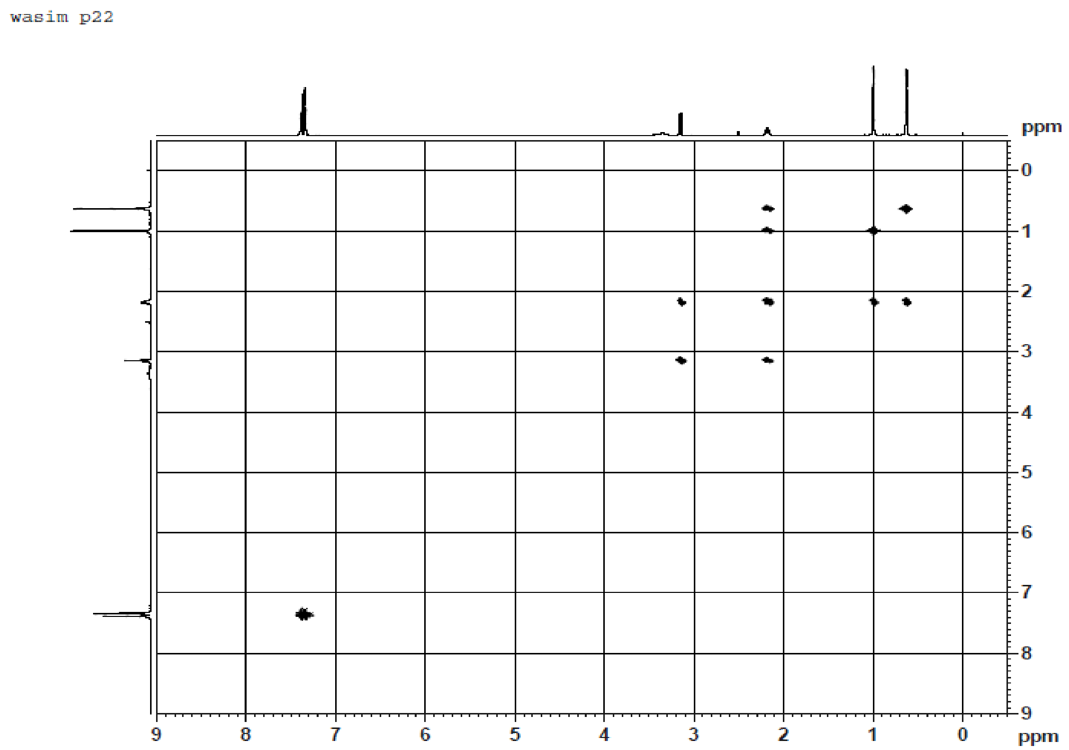

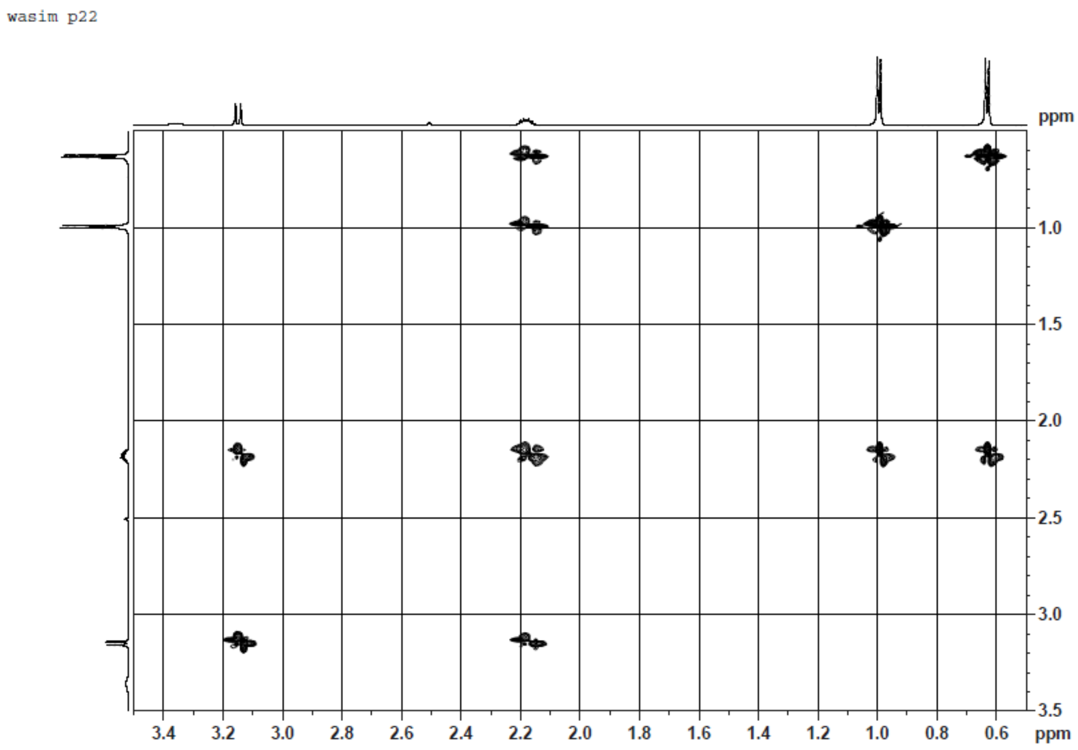

Figure S12: COSY correlation of compound **XII**

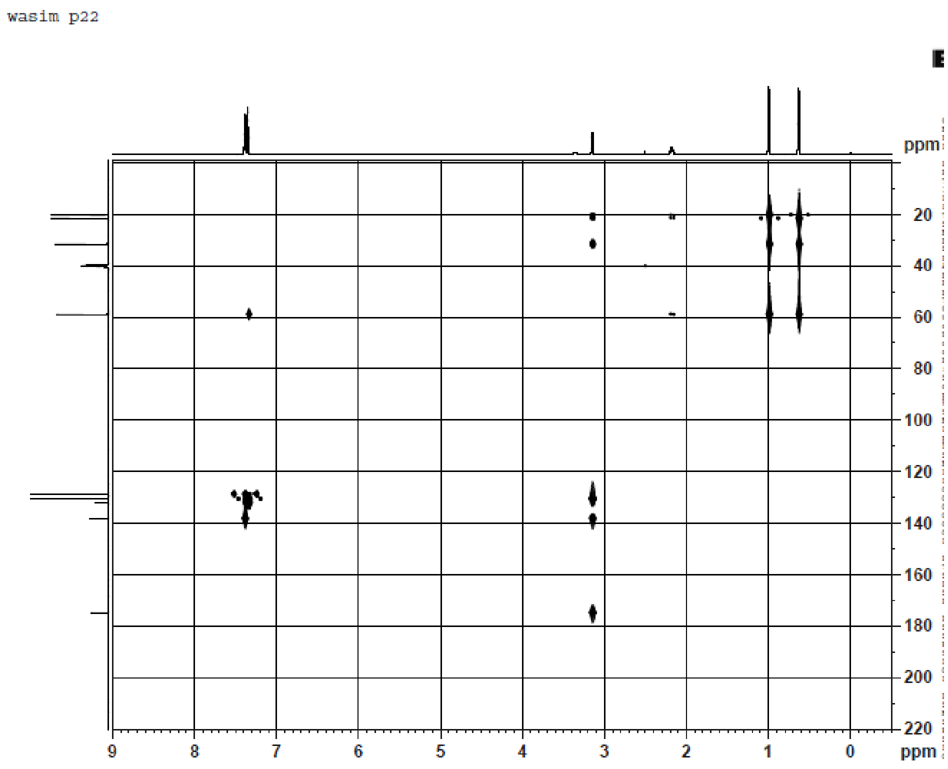

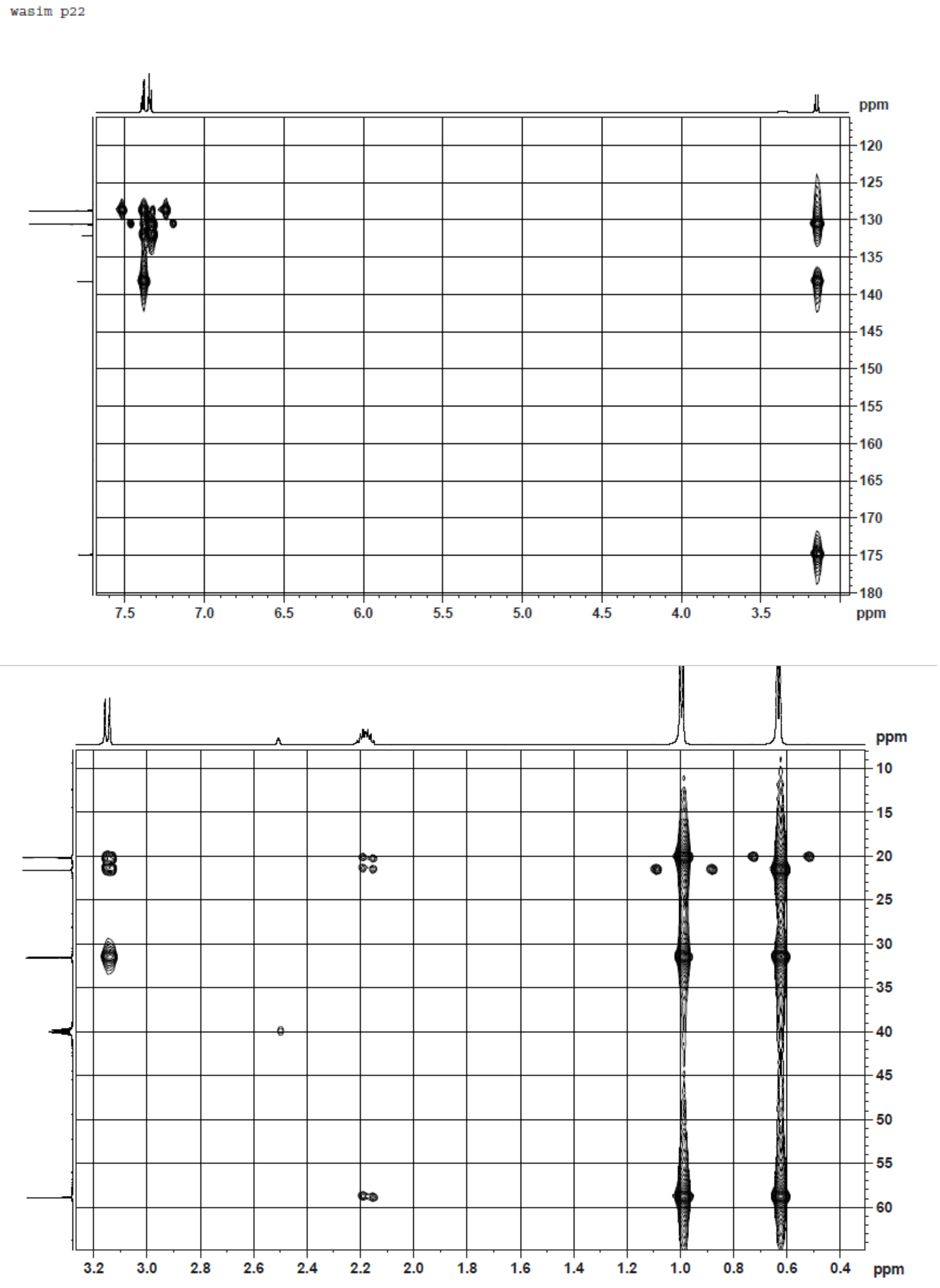

Figure S13: HMBC correlation of compound **XII**
